# The History, Mechanism, and Clinical Application of Auricular Therapy in Traditional Chinese Medicine

**DOI:** 10.1155/2015/495684

**Published:** 2015-12-28

**Authors:** Pu-Wei Hou, Hsin-Cheng Hsu, Yi-Wen Lin, Nou-Ying Tang, Chin-Yi Cheng, Ching-Liang Hsieh

**Affiliations:** ^1^Department of Chinese Medicine, China Medical University Hospital, Taichung 40447, Taiwan; ^2^Graduate Institute of Acupuncture Science, College of Chinese Medicine, China Medical University, Taichung 40402, Taiwan; ^3^School of Chinese Medicine, College of Chinese Medicine, China Medical University, Taichung 40402, Taiwan; ^4^Graduate Institute of Integrated Medicine, College of Chinese Medicine, China Medical University, Taichung 40402, Taiwan

## Abstract

Auricular therapy includes acupuncture, electroacupuncture, acupressure, lasering, cauterization, moxibustion, and bloodletting in the auricle. For 2500 years, people have employed auricular therapy for treating diseases, but the methods have been limited to bloodletting and cauterization. Only after 1957, the international scientific community became aware that the map of the ear resembles an inverted fetus, its introduction has led to auricular acupuncture (AA) becoming a more systemic approach, and, following the identification and standardization of more precise points, AA has been employed in clinical applications. The mechanisms of AA are considered to have a close relationship with the autonomic nervous system, the neuroendocrine system, neuroimmunological factors, neuroinflammation, and neural reflex, as well as antioxidation. Auricular therapy has been applied, for example, for pain relief, for the treatment of epilepsy, anxiety, and obesity, and for improving sleep quality. However, the mechanisms and evidence for auricular therapy warrant further study.

## 1. Introduction

Auricular acupuncture (AA) is a method for diagnosing and treating physical and psychosomatic dysfunctions by stimulating a specific point in the ear [1]. Ear stimulation involves the neurological reflex, neurotransmitters, cytokines, the immune system, and inflammation [1–3]. AA has been employed for approximately 2500 years, for which the oldest record is* Huang Di Nei Jing* (*The Yellow Emperor's Classic of Internal Medicine*), written in Chinese, and a report by Hippocrates is the oldest Western record [4]. In Traditional Chinese Medicine (TCM), the ear is directly or indirectly connected with 12 meridians, and stimulating the ear can restore the balance between Qi and blood [5]. In Europe, AA has been applied systematically and comprehensively since Doctor Nogier introduced the inverted fetus map in 1957 [6–9]. This article by Nogier was read by Russian, Japanese, and Chinese acupuncturists and was translated in 1958 and 1959 into Chinese, the latter reporting only Nogier's original image of the somatic parts of the body [10]. Information regarding the entire organism or body part can be retrieved from the ear (i.e., the holography rule of points) [11]. Various methods currently existing for ear stimulation are needles, seeds, magnetic stones, lasers, ultrasound, bloodletting, moxibustion, electric treatment, and pressure by hands [4, 12]. AA is a convenient and basic method used for treating many conditions (e.g., substance abuse, pain, obesity, anxiety, epilepsy, and sleep disorders), but the effectiveness of AA has been tested only in a relatively small number of evidence-based trials [5, 13]. Gueguen et al. (2013) reported a system review about 42 randomized control trials. AA has the effect to decrease the preoperative anxiety and peroperative pain but no effect in the prevention of withdrawal syndrome according to the poor methodological studies [14]. With technological advancements, increasingly more clinical trials conducted in the field of biochemistry are presenting evidence regarding the detailed mechanisms of AA in the treatment of diseases. Therefore, this study traces and presents the history, mechanisms, and the clinical application of AA.

## 2. History of Auricular Acupuncture

### 2.1. In Europe and Surrounding Regions

In Europe and surrounding regions, AA has been applied for approximately 2500 years. Numerous historical records of AA are found worldwide. In Egypt, women can cauterize and stick a needle in the ear for sterilization. In Italy, the wounded of the auricle in war could occasionally heal their original ailments. In Saudi Arabia, certain tribes cauterize the ear to heal diseases [1]. The Mediterranean peoples wear an earring for improving their eyesight [4]. In Greece, the first record (ca. 460 BC) of AA is a report by Hippocrates, which stated that phlebotomy (i.e., applying a needle to the ear to create an incision in a vein) was conducted for treating impotence and facilitating ejaculation [4]. Another source indicated that phlebotomy can relieve leg pain [1]. The Roman Empire received medicine from Ancient Greece, present-day Egypt, Persia, and present-day Saudi Arabia, and they still use these methods for treating sciatica, hip pain, and sexual ailments [1]. In 1637, a physician named Zacatus Lusitanus cauterized the ear to treat sciatica after phlebotomy did not produce the desired effect. In 1717, the physician Antonio Maria Valsalva discovered a new point that can alleviate odontalgia after cauterization. In 1810, a professor named Ignazio Colla used a bee needle to stimulate the ear and relieved pain in the lower extremities. After 1850, numerous reports described major cases of ear cauterization conducted for tooth extraction. Journal des Connaissances Medico-Chirurgicales, a French journal, mentioned 13 cases of patients with sciatica treated with ear cauterization [1, 4]. In 1957, Doctor Paul Nogier reported the somatotopic correspondence in the ear, which marked the emergence of a new era for AA application.

### 2.2. In China

**Figure 1:**
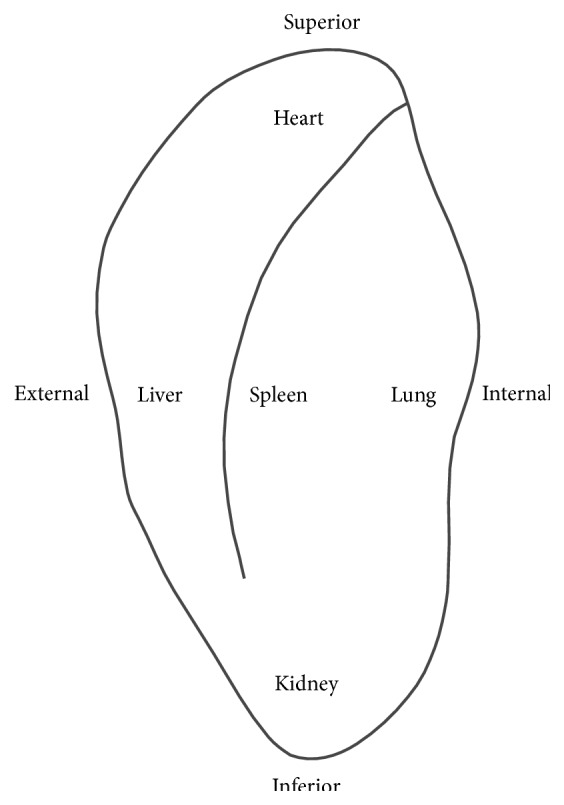
The five viscera distribution of ear in Traditional Chinese Medicine recording.

The oldest record on AA is from* Huang Di Nei Jing* (ca. 100 BC) [1]. Chapter 63 in the plain questions of* Huang Di Nei Jing* stated that a physician can employ a tube and blow air into the ear to save an unconscious patient. Chapter 20 in the miraculous pivot of* Huang Di Nei Jing* indicated that phlebotomy in the distended vein can relieve the tightness in the costal regions.* Zhou Hou Bei Ji Fang* (ca. 300 to 400 AD), emergency handbook, recorded that using aconite-derived oil, gladiolus, and sesame oil to irrigate the auditory meatus can treat ear pain.* Qian Jin Yao Fang* (652 AD), authored by Sun Si-Miao, stated that the point in the helix before the concha ridge can be targeted for treating jaundice. In* Wei Sheng Bao Jian* (1343 AD), Luo Tian-yi wrote that cauterizing a vein behind the ear can treat the infantile convulsion. In* Zhen Jiu Da Cheng* (1601 AD), an acupuncture text composed by Yang Ji-Zhou indicated that performing phlebotomy inside the tip of the ear can be effective for treating eye diseases. Concerning AA in the treatment of specific organs,* Essential Techniques of Massage* (Chinese name,* Li Zheng An Mo Yao Shu*, 1888 AD) written by Zhang Di-Shan divided the auricle into five regions, with each, respectively, targeting the heart, lung, liver, spleen, and kidney, and indicated that a functional organ can be identified by noting any changes in these respective regions (Figure 1) [15–17].

### 2.3. Modern Period

In 1956, Doctor Paul Nogier, the father of AA, presented his inverted fetus map at the congress of the* Société Mediterranéenne* in Marseille [4]. Nogier had found that sciatica could be healed by the cauterization in the inferior crus of the antihelix. He conducted repeated investigations into the conditions before introducing 37 AA points [1, 4, 18]. In 1966, he found that changes to the pulse rate in the radial artery were related to the stimulation of the auricle and coined this phenomenon “*Reflexe Auriculocardiaque*” (Vascular Autonomic Signal, VAS) [1, 4, 19]. After a journal published this finding, the Nanjing Army Ear Acupuncture research team recruited over 2,000 patients to establish an AA model, and their results ultimately confirmed Nogier's propositions from 1958 [1, 4, 5, 13, 20]. More than 1,000 AA acupoints have since been identified, and, in 1982, the Western-Pacific Regional Office, World Health Organization (WHO), convened a conference to discuss how to eliminate the confusion originating from duplicate names and regions. Finally, in 1987, the WHO released a report entitled “Scheme of Standardization of Auricular Acupoints” [1, 4, 5]. The standardization process was afterwards carried on only by Chinese researchers and the final document on nomenclature and location of ear acupuncture points was published in 1993 and confirmed in 2008 [17]. Actually, the use of acupoints in Europe still differs from Chinese practice, but it has to be pointed out that the current Chinese maps correspond faithfully to the historic maps of Nogier published in 1957 [18].

## 3. Mechanisms of Auricular Acupuncture

### 3.1. Anatomy of the Auricle

**Figure 2:**
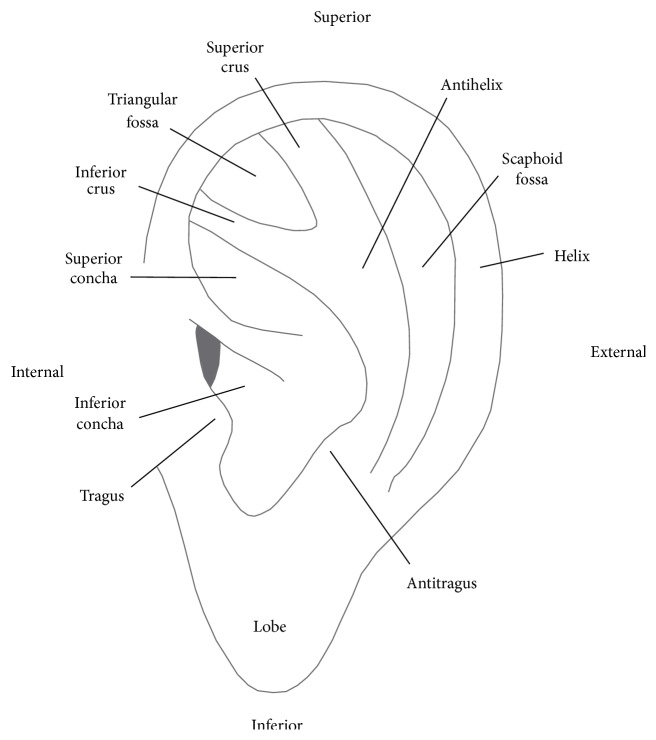
Anatomical structure of ear.

The basic terminology regarding the auricle concerns the prominent parts (i.e., the helix, antihelix [including the superior and inferior crus], and the tragus and antitragus); the concave parts (i.e., the scaphoid fossa, triangular fossa, and the superior and inferior concha); and a flat part (i.e., the lobe; Figure 2) [13, 21]. The ear is innervated by cranial and spinal nerves, which are separated into motor and sensory areas. The motor area concerns the motor branch of the facial nerve (CN VII), which controls the outer ear muscles. The sensory area is composed of auricular branches of the vagus nerve (ABVN), the auriculotemporal nerve (a branch of CN V), the sensory area of the facial nerve, the glossopharyngeal nerve, the lesser occipital nerve, and the greater auricle nerve. The most important nerve is ABVN because of its function in AA in affecting mainly the concha and most parts of the auditory canal [4, 9].

### 3.2. Theory of Auricular Acupuncture

#### 3.2.1. Somatotopic Arrangement (Homuncular Theory)

**Figure 3:**
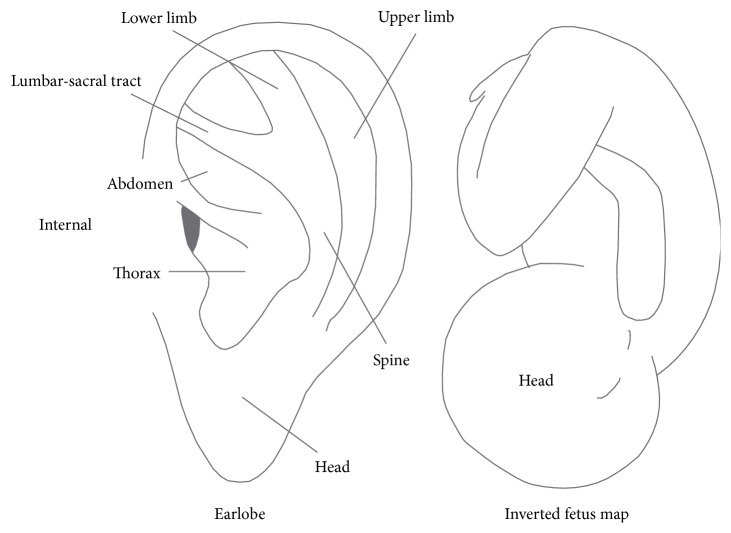
Ear map as like an inverted fetus.

Nogier had devised the map of an inverted fetus by noting its resemblance to the ear, and this map is the most widely used reference for diagnosing and treating auricular diseases (Figure 3) [1, 4, 13]. The general arrangement is that the earlobe targets the head and brain, the antihelix represents the spine, the scaphoid fossa refers to the upper extremities, the superior and inferior crus target the lower extremities, and the concha represents the internal organs, but numerous differences still exist between the Europe and Chinese systems [1, 4, 13]. Nogier believed that the relationship between AA and the areas of the body is due to the vagus nerve-autonomic nervous system (ANS) [1, 4, 13]. Doctor Oleson, a US physician, used somatotopic arrangement of the auricle to diagnose the medical condition with a 75.2% accuracy rating [1]. Andersson et al. (2007) recruited 25 patients with chronic pain in a double-blind study to examine whether auricular maps correspond to parts of the body but they found that the AA map was not corresponding to the body parts [22]. Another blinded study was made by Romoli et al. on 506 patients employing 3 different diagnostic methods: inspection of the auricle, pain pressure test (PPT), and electrical skin resistance test (ESRT). Inspection was superior to PPT and ESRT and applying all three diagnostic methods together can achieve a success rate of 78.6% in identifying symptoms and syndromes related to somatic and mental disorders [23, 24].

#### 3.2.2. Embryological Regions

Embryological organization is similar to somatotopic organization. The earlobe and tragus correspond to the ectoderm, the concha corresponds to the endoderm, and the remaining portion of the ear corresponds to the mesoderm. If an organ (e.g., the lungs) corresponds to the endoderm, we can use the concha to treat or diagnose the condition [4, 5, 25]. The distribution of embryological regions is based on the lower portion of the ear representing the head and the upper portion representing the foot [13].

#### 3.2.3. Meridian Theory

According to meridian theory, the ear is connected directly or indirectly to 12 meridians. The* miraculous pivot* of* Huang Di Nei Jing* indicated that any channel and meridian converge in the ear [4, 15]. The application of AA in TCM is based on yin-yang theory and five-element theory, but most auricular treatments involve the acupoints of the four limbs and the visceral and endocrine related areas innervated by vagus nerve [26]. Regarding the application of AA in Ancient China, their traditional methods could not be devised into a theoretical framework and are akin to home remedies.

### 3.3. Mechanisms of Action

#### 3.3.1. Connection between the Auricle and Autonomic Nervous System

In 1832, Friedrich Arnold, a German professor of anatomy, found that stimulating the external ear canal can induce a cough similar to the cough reflex induced by the vagal nerve. This reflex is called “Arnold's Reflex” and regards the ABVN as its afferent nerve [9]. Because ABVN stimulation can induce a response similar to that of the vagal nerve, the ABVN may have a relationship of the auricle and ANS [9].

The ABVN delivers fiber into the nucleus of the solitary tract (NTS). Nomura and Mizuno (1984) applied horseradish peroxide to the end of the ABVN in cats and found that the labeled nerve fibers of the ABVN surround the NTS [27]. The inputs of the NTS include fibers from the facial nerve (CN VII), the glossopharyngeal nerve (CN IX), the vagus nerve (CN X), and afferent nerves in the internal organs. By contrast, the NTS outputs include reticular formation, parasympathetic preganglionic neurons to the viscera, the paraventricular nucleus of the hypothalamus, the thalamus (visceromotor center), and amygdala. The NTS mediates many reflexes, for instance, the carotid sinus reflex (chemoreceptor and mechanoreceptor), the aortic reflex (chemoreceptor and mechanoreceptor), the gag reflex, and the cough reflex, as well as several respiratory and gastrointestinal reflexes regulating organ function [28]. The concha of the auricle and the external auditory canal, especially the inner part of the tragus, is supplied mainly by the ABVN. Stimulation originates from the cutaneous concha through the auricular nerve (CN IIX) and travels to the jugular ganglion, and the branches of the vagus nerve start from this ganglion and end in the NTS of the medulla oblongata [29]. Based on the complex connections in the NTS between the brain and the viscera, ABVN stimulation can regulate the ANS.

The stimulus from AA raises the vagal tone and regulates the cardiovascular, respiratory, gastrointestinal, and endocrine systems [9]. Regarding the cardiovascular system, AA can lower the heart rate and blood pressure and accelerate blood flow and heart rate variability (HRV) [30]. Moreover, stimulating the heart acupoint of the ear in rats was found to lower arterial pressure and the heart rate significantly compared with the use of Zusnali (ST36) and Neiguan (PC6) [31]. Regarding the respiratory system, AA combined with electrical treatment had a positive effect on respiratory sinus arrhythmia by increasing vagal activity [32]. For the gastrointestinal system, the effects of AA on motility and the gastrointestinal tone were similar to those from medication [33].

#### 3.3.2. Delta Reflex Theory

Delta reflex theory posits that cold or heat stimulation on parts of the body raises the temperature in the corresponding parts of the ear from 1.0°C to 5.5°C. Doctor Cho proposed this theory in the 1970s, which posits a relationship between parts of the body and the regions of the ear [5]. This reflex may be influenced in part by the vagal nerve.

#### 3.3.3. Acupoints in the Ear: Experiences with Functional Magnetic Resonance Imaging (fMRI)

Gao et al. (2008) proposed that stimulating different points in the ear can induce a similar response on the cardiovascular and gastrointestinal systems [34]. Alimi et al. (2002) demonstrate that acupuncture in the point of the ear for the hand leads to selectively altered fMRI changes in the somatosensory cortex for the hand of the postcentral gyrus [35]. Romoli et al. (2014) employed fMRI to detect the differences between two stimulated acupoints: Thumb Auricular Acupoint (TAA) to bilateral parietal operculum and the second somatosensory area and Brain Stem Auricular Acupoint (BSAA) to the limbic and cortical areas [36]. Alimi et al. (2014) used fMRI to prove the topography of the French-German auricular area better than the Chinese auricular area [37].

#### 3.3.4. Anti-Inflammation

Ceccherelli et al. (1999) used carrageenan injection to make the inflammation of the rat's paw and the real electroacupuncture of AA has the anti-inflammatory effect [38]. Chung et al. (2011) found the same effect like above but the mechanism of action was blocked by methyl atropine which inhibits cholinergic muscarinic receptor—not naloxone, an inhibitor of the systemic opioid receptor [39]. Zhao et al. (2012) reported that transcutaneous auricular vagus nerve stimulation (ta-VAS) has an effect similar to that of vagus nerve stimulation (VNS). VNS regulates the immune system via the cholinergic anti-inflammatory pathway. The researchers administered rats with lipopolysaccharides through intravenous injection to induce inflammation. They found that VAS and ta-VNS reduced the serum levels of proinflammatory cytokines in lung tissue. The influence of ta-VNS can be suppressed by performing vagotomy or with the *α*7nAChR (nicotine acetylcholine receptor) antagonist [2]. Ceccherelli et al. suggest that superficial acupuncture is a real placebo method and the level of the pain threshold depends on the intensity of stimulation. They found that an “afferent” somatotopic representation is not equally an “efferent” somatotopy. The electrical stimulation of the ear decides the increased level of the pain threshold in the whole body and not only in the zone somatotopically according to the auricular acupoint [40].

#### 3.3.5. Antioxidation

Liu et al. (2008) recruited 69 patients with high-risk diabetes mellitus and stimulated the shenmen as well as the kidney and endocrine acupoints for 20 days and found a significant reduction in serum superoxide dismutase (SOD) and catalase concentrations [41].

## 4. Clinical Application

### 4.1. Analgesia

AA is applied for managing various types of pain such as postoperative, dental, and musculoskeletal pain, as well as pain related to anesthesia [42]. The analgesic effects of AA are induced by activating the descending pain inhibitory pathway of the brainstem, thereby inhibiting the ascending pain pathway. AA application can activate the descending pain inhibitory pathway along the dorsal side of the spinal cord where the dorsal horn cells are located, which exert a pain-relieving effect. Thus, deep brain stimulation can produce analgesic effects by suppressing the dorsolateral funiculus in the spinal tract. Nociceptive pain can trigger activity in the hypothalamus, periaqueductal gray, somatosensory cortex, and prefrontal cortex, but deep brain stimulation can also activate the same regions in the subcortical thalamus to produce analgesic effects. This stimulation-induced analgesic effect increases the concentration of beta-endorphins and can be blocked by naloxone [43].

Simmons and Oleson (1993) conducted a study to measure changes in dental pain after treatment. The group of auricular electrical stimulation after saline injection had an elevated pain threshold of over 23% but the effect is reduced to less than 12% by naloxone. The analgesic effect from AES does not originate only from the endogenous opioid system [44]. Oliveri et al. (1986) used high-intensity transcutaneous electrical nerve stimulation (TENS) on the acupoints of the ear to increase pain threshold [45]. Woodward Krause et al. (1987) found that electrical stimulation (low frequency, 1 Hz, and high intensity, 1000 *μ*A electrical stimulation) on one or two ears can elevate the pain threshold [46]. Noling et al. (1988) found that a low frequency (1 Hz) with a high intensity (1000 *μ*A) elevated the pain threshold, and this effect peaked 5 to 10 min after stimulation and lasted from several hours to a few days [47]. Lein et al. (1989) used TENS and also had the same result as above [48]. Sator-Katzenschlager et al. (2003) found that auricular electrical stimulation is better than auricular manual acupuncture when decreasing pain [49]. Sator-Katzenschlager et al. (2004) obtained similar findings for auricular EA [50]. Yeh et al. (2014) found an average reduction of 63% in pain intensity at Day 7 in the 27 participants who completed the 4-week treatment [51].

Taguchi et al. (2002) found that AA reduced the anesthetic (i.e., desflurane) requirement about 8.5% [52]. Greif et al. (2002) found that AES reduced the need about 11% [53]. Kindberg et al. (2009) reported that AA application led to greater pain relief compared with local anesthesia (53% versus 19%, resp.) after postpartum surgical repair [54]. Wetzel et al. found that AA reduced the requirement of fentanyl by 15% in 120 patients who underwent total hip arthroplasty [55]. He et al. (2013) employed the four acupoints of the ear (i.e., knee, shenmen, subcortex, and sympathesis) to reduce postoperative pain and the need of anesthesia after total knee arthroplasty [56].

About migraine, Romoli et al. used AA to treat migraine attacks and an innovative diagnostic test called needle-contact test (NCT) or use semipermanent needles to maintain the effect [57, 58]. Allais et al. (2011) found that using “appropriate” points is better than “inappropriate” points when improving migraine and the appropriate points are corresponding to the somatotopic representation of our body on the ear [59].

The application of ear acupuncture in treating postoperative pain remains controversial [60–65]. Usichenko et al. (2005, 2007) found that AA can reduce the consumption rate of ibuprofen in the 120 patients who underwent ambulatory knee surgery [60, 62]. Moreover, Usichenko et al. (2005) found a reduced consumption rate of piritramide in the patient who received total hip arthroplasty [61]. Michalek-Sauberer et al. (2007) found that the required acetaminophen dosage did not differ significantly among the 3 groups (auricular EA, AA, and sham auricular EA) [63]. Yeh et al. (2010) reported that auricular acupressure cannot relieve pain, reduce the dose of analgesics, or alleviate nausea and vomiting in the postoperative patient [65]. Regarding low back pain (LBP), AA has been found to be a beneficial and safe treatment [66–68]. Suen and Wong (2008) found that the longitudinal effects of AA in the elderly with LBP are improving the disability level, pain and sensation, and functional activity [66]. Wang et al. (2009) found that there are significant differences in pain relief of AA in 311 pregnant women with LBP [67]. Hunter et al. (2012) found that the patients in the exercise with AA group reported greater changes in the Oswestry Disability Questionnaire (of roughly 10.7% in points) compared with the exercise-only group at the end of the 6-month follow-up period [68]. Romoli et al. (2014) found that, after one session of ear acupuncture in a group of total knee replacement patients, AEA can reduce pain in sitting and standing position and improve sit-to-stand performance and these variations are still significantly maintained for six hours [69].

Regarding cancer pain, Dillon and Lucas (1999) found that AA led to rapid decreases in pain scores, and the treatment effect was sustained over a long period (i.e., 4 weeks) [70]. Alimi et al. (2003) found that the level of cancer pain decreased 36% in the auricular acupuncture group and auricular EA group but there was less change for patients in the placebo group [71]. Asher et al. (2010) collected 17 randomized control trials in their review and only included 12 studies in their meta-analysis. They found that AA is effective for pain relief but needed more accurate evaluations and a rigorous methodology [72]. Yeh et al. (2014) conducted a review and found that EA in the ear did not yield significant improvements in pain management [42]. Large-sample studies and a strong methodological design are required in order to discern the true efficacy of AA.

### 4.2. Epilepsy Treatment

Epilepsy is a disease inducing repeated seizures triggered by excessive and abnormal brain activity. The antiepileptic effect of AA is related to the vagus nerve and NTS. Auricular stimulation sends signals through the ABVN into the NTS and reaches other parts of the brainstem, hippocampus, thalamus, and amygdala. The signals from the NTS regulate inflammation by adjusting the levels of TNF-*α* and IL-1*β* in the brainstem and hippocampus, thereby preventing the thalamus and amygdala from inducing sleep as well as increasing kindling resistance [73]. He et al. (2013) found that ta-VNS significantly increased the first grand mal latency and the firing rates of the NTS that can suppress epileptiform activity; the reversible cold block of the NTS can inhibit the anticonvulsant effect [74]. Lin and Hsieh (2014) examined the effects of AA and somatic acupuncture in rats with kainic acid- (KA-) induced epilepsy. EA (ST36-ST37, 2 Hz) can decrease hyperexcitability in the brain of rats. Transient receptor potential ankyrin 1 (TRPA 1), which regulates inflammation resulting from environmental exposure, can be controlled by extracellular signal-regulated kinase (ERK), protein kinase A, and protein kinase C (PKC). Auricular EA can reduce epileptiform discharge and the levels of pPKC*ε*, pPKC*α*, and pERK1/2 and prevent brain inflammation, which leads to epileptic seizures [3]. Liu et al. (2014) found the association between mossy fiber spouting which can be increased by KA contributing to epileptogenesis and auricular EA and ST36-ST37 and AA can reduce the formations of mossy fiber spouting in rats [75]. More randomized clinical and control trials are required to further our understanding regarding the exact effects of AA treatment in patients with epilepsy.

### 4.3. Substance Dependence

Regarding cocaine dependence, AA is an adjunct treatment combined with conventional approaches including relaxation, medication, and counseling. Wen and Cheung (1973) stated that auricular electrical acupuncture (AEA) in the patient with addiction can improve the withdrawal symptoms [76]. Ng et al. (1975) described that AEA can treat withdrawal syndrome but this effect was inhibited by naloxone. AEA treatment can produce significant decrease of certain naloxone-precipitated morphine abstinence signs in rats [77, 78]. The National Acupuncture Detoxification Association (NADA) treatment protocol has been the most applied protocol since 1977 [79]. Cocaine can inhibit the reuptake of neurotransmitters in the brain, especially that of dopamine. AA can activate the neuronal release of serotonin in the hypothalamus via the ABVN. Serotonin can activate met-enkephalin, which can inhibit the release of *γ*-aminobutyric acid (GABA). GABA can inhibit dopamine output in the brain. Finally, AA can increase dopamine levels by inhibiting GABA [80]. Margolin et al. (1996) found that the preselection of acupoints in the ear is not an effective approach for treating subjects with cocaine dependence [81]. Bullock et al. (1999) revealed a nonsignificant difference among the groups as well as in the varied number of AA treatment sessions [82]. Margolin et al. (2002) found that there is a significant reduction in cocaine use but no differences among AA group, a needle-insertion control group, and a relaxation control group [83]. Killeen et al. (2002) found no differences in the psychological and physiological effects of AA between AA and sham AA groups [79]. D'Alberto (2004) conducted a review and found randomized control trials of adequate methodological quality but still could not support the NADA protocol in treating cocaine dependence [80]. Gates et al. conducted a Cochrane review and collected seven studies (comprising 1,443 participants) with low methodological quality, and the conclusions on this issue were inconsistent because of the small samples [84]. Janssen et al. (2012) evaluated the effect of AA (NADA protocol) in pregnant women with drug dependence and found that AA did not have benefit more than the control group. However, the use of methadone may induce an adverse event in the fetus, and AA may be a safe method [85].

Regarding smoking cessation, the mechanism of action of AA is related to the dopamine concentration in the brain. One study found a genetic variation between high and low responders after AA treatment; specifically, the detection frequency of the dopamine D2 receptor^*∗*^A1 allele (DRD2 TaqI A) was lower in high responders. Detection of the DRD2^*∗*^A1 allele is related to fewer dopamine receptors [86]. Waite and Clough (1998) recruited 78 smokers into the trial and only 5 participants in the AA group can keep cigarette away after 6 months [87]. Bier et al. (2002) combined AA and education on smoking cessation and cigarette consumption and revealed a significant reduction in smoking cessation and cigarette consumption in AA combined with education group. Moreover, the treatment effect had a negative correlation with the pack-year history [88]. Another study on AA with an Internet-assisted smoking cessation program also found a significant effect compared with AA only [89]. Wu et al. (2007) found a significant reduction in cigarette consumption in both groups (AA and sham group), and the AA group lowered nicotine withdrawal symptom scores. Moreover, no differences were found in the smoking cessation rate in both groups at the end of treatment as well as the follow-up [90]. Yeh et al. (2014) recruited 96 college students and assigned them to AA, interactive media (IM), and control groups. The authors evaluated FEV1, carbon monoxide (CO), and cotinine levels as well as nicotine dependence before and after the 10-week intervention period and found a significant reduction in CO levels and nicotine dependence in all 3 groups as well as lower cotinine levels in the AA and IM groups. However, no differences in FEV1 levels were found in all 3 groups before and after the intervention [91]. White and Moody (2006) investigated the effects of correct and incorrect acupoints on smoking cessation and identified 10 studies (four with high validity) indicating that the correct acupoints were more effective than the incorrect acupoints. Moreover, three high-quality studies reported no differences in the effect of AA between correct and incorrect acupoints, whereas two other studies indicated that incorrect acupoints had greater efficacy compared with correct acupoints. Based on these findings, they concluded that AA efficacy may not be correlated with the position of acupoints [92]. Di et al. (2014) evaluated two issues related to smoking cessation: (a) differences in efficacy between specific ear therapy (including acupuncture, acupressure, and auriculotherapy) and nonspecific control therapy and (b) differences in efficacy between specific ear therapy and specific treatments (i.e., behavioral therapy and body acupuncture). Their results showed that specific ear therapy was better than nonspecific control group and showed no differences between specific ear therapy and specific treatment [93]. The findings confirmed the efficacy of AA in smoking cessation, but the selected acupoints remained controversial.

For alcohol dependence, AA treatment has been found to be ineffective. Sapir-Weise et al. (1999) found no differences between correct and incorrect points in the drinking days and craving status. However, they noted a reduction in anxiety among women who received treatment with correct acupoints at 1-month follow-up [94]. Bullock et al. (2002) showed the significant improvements in the depression, anxiety, and functional status in all groups but no differences in the specific acupuncture, nonspecific acupuncture, and symptom-based acupuncture groups [95].

### 4.4. Antipsychogenic Effect

Patients may experience fear and anxiety before surgery, and this psychological state may complicate the induction of anesthesia, leading to a poor disease outcome [96]. Romoli and Giommi (1993) identified the Triple Heater (TH,* Sanjiao*) area of the Chinese map as the major area for diagnosing and treating a stress response related to important life events in order to reduce anxiety and depression [26]. Wang and Kain (2001) found that relaxation group is better than shenmen and sham groups in the State-Trait Anxiety Inventory State Scale (STAI) in the chronic disorder [97]. Wang et al. (2001) evaluated the changes in STAI scores in preoperative patients after receiving AA (based on TCM) relaxation, and control groups. The results revealed that the relaxation group had significantly lower STAI scores than the control group, but not the AA group [11]. Kober et al. (2003) evaluated the effect of AA (relaxation group) in alleviating anxiety in the ambulance. The relaxation group lowered the scores of the anxiety and anticipated pain measured by the Visual Analog Scale and improved the disease outcomes. The mechanism may induce the release of endorphins and neurotransmitters, serotonin, norepinephrine, and GABA in the brainstem, midbrain, and hypothalamus [98]. Wang et al. (2004) investigated the effect of AA on mothers and their children scheduled for surgery. The mothers and their children in the AA group reported a significant reduction in STAI scores and on the modified Yale Preoperative Anxiety Scale compared with the control group. Moreover, children in the AA group reported higher scores in the Induction Compliance Checklist compared with the control group [96]. Mora et al. (2007) examined the effectiveness of AA in alleviating anxiety in patients before receiving extracorporeal shock wave lithotripsy. The results revealed a significant reduction in anxiety, reduction in pain, and improved treatment outcome in the AA group [99]. Karst et al. (2007) investigated the effectiveness of AA versus medication treatment on dental anxiety. The results revealed that AA and intranasal midazolam had similar effects in alleviating anxiety and raising compliance [100]. Black et al. (2011) examined the efficacy of AA in patients withdrawing from psychoactive drugs. The NADA protocol group did not report higher scores in reduced anxiety compared with the sham AA and control groups [101]. Michalek-Sauberer et al. (2012) examined AA efficacy in patients with dental diseases. The AA group reported lower anxiety scores compared with the sham group, whereas the control group reported increased anxiety [102]. Reilly et al. (2014) investigated the efficacy of AA on anxiety levels in caregiver. The results revealed a significant decrease in STAI scores and higher Caring Ability Inventory scores in the AA group [103]. Gagliardi et al. (2014) enrolled 20 health volunteers (divided into real and sham groups) and assessed the anxiolytic-sedative effect of AA on health person. There was a significant reduction of the numeric rating scale anxiety score (*p* < 0.01) and State-Trait Anxiety Inventory State anxiety score values (*p* < 0.005) in the real acupuncture group. The Bispectral Index System score did not change after 5 minutes, but a significant decrease in anxiety was noted in the real acupuncture group [104].

Regarding patients with depression, Nixon et al. (2003) examined the efficacy of AA in treating depression in adolescents with repetitive self-injurious behavior (SIB). The results revealed a significant decrease in SIB frequency 4 weeks after treatment, although the decrease in the urge to self-injure was nonsignificant [105]. Shi et al. (2013) examined the effects of continuous auricular EA in patients with depression. The results revealed a significant increase in HRV as well as lower scores on the Hamilton Anxiety Rating Scale (HAM-A), the Athens Insomnia Scale (AIS), and the Hamilton Rating Scale for Depression (HRSD), but the changes in heart rate and low frequency/high frequency ratio (LF/HF ratio) were nonsignificant [106]. Liu et al. (2013) investigated the efficacy of auricular EA in rat models. The findings revealed a significant reduction in blood pressure comparable to that achieved through VNS as well as a significant decrease in HRV in the EA in the auricular concha region (EA-ACR) group, but not in the other groups. EA-ACR groups experienced a significant reduction in plasma cortisol and adrenocorticotropic hormone (ACTH) levels. The effect of EA-ACR on depressed rats may be induced by the normalization of hypothalamic-pituitary-adrenal axis hyperactivity [107].

### 4.5. Insomnia

Insomnia affects many people of all ages and contributes to many disorders (e.g., fatigue, instability, depression, impaired daily function, anxiety, and substance abuse) [108]. Insomnia is diagnosed when the quality and amount of sleep are deemed unsatisfactory, and when people have difficulty falling asleep, staying asleep, and waking up too early [109]. The mechanism of AA in insomnia may involve the regulation of melatonin [110]. Suen et al. (2002) evaluated the effects of auricular therapy on sleep promotion. The results revealed significant improvements in nocturnal sleep time and sleep efficiency in all groups, but significant improvements in sleep behavior were found only in the magnetic pearl group [111]. One year later, Suen et al. (2003) reported long-term effects for magnetic pearl auricular therapy in treating insomnia in elderly people after identifying significant changes in nocturnal sleep time and improvements in sleep behavior in 15 elderly patients, and this effect was retained 6 months after treatment [112]. Kim and Sok (2007) examined the efficacy of AA in treating insomnia. The sleep state and sleep satisfaction improved significantly, and the effect lasted for 2 weeks [109]. Sjöling et al. (2008) examined the effects of AA on patients with insomnia. The results revealed nonsignificant differences in total sleep time in both groups, as well as in the frequency of waking up and ease in waking up in the AA group. However, the other sleep parameters improved substantially in both groups during treatment [113]. Wu et al. (2014) conducted a pilot study to evaluate the effects of AA on hemodialysis patients with insomnia. The results revealed significant decreases in the scores of the Pittsburgh Sleep Quality Index (PSQI) for sleep quality, sleep latency, sleep disturbance, daytime dysfunction, and reduced intake of medication [114]. Review articles were consistent in their conclusion that AA had an effect on insomnia, but the low methodological quality of these studies limited the validity of their findings [12, 108, 115, 116].

### 4.6. Obesity

Obesity was found to raise the risk of metabolic syndrome and cardiovascular and cerebrovascular diseases [117, 118]. The causes of obesity included an unbalanced diet, genetic heredity, socioeconomic factors, endocrine diseases, lack of exercise, and emotional issues [118]. Asamoto and Takeshige (1992) [119] stimulated the rat inner auricular areas that represent the human pylorus, lung, trachea, stomach, esophagus, and endocrine, and heart acupuncture points induced the action potentials in the hypothalamic ventromedial nucleus (HVM), the satiety center. Needle implantation into any of these points reduced the body weight of rats. Stimulation of other acupuncture points did not induce HVM action potentials. If the HVM was destroyed, the AA had no effect on body weight. There were no effects of the AA on the lateral hypothalamus (LHA). Shiraishi et al. (1995) investigated whether the electrical auricular stimulation affected the activities of the LHA and HVM in rats. The results revealed reduced activity in LHA neurons and increased activity in VMH neurons after electrical auricular stimulation. Even the nonobese rats still have the effects of the AA on reducing body weight [120–122]. Kim et al. (2001) found that AA treatment in unfed rats can lower neuropeptide Y (NPY), whereas in fed rats it can increase NPY [123]. Cabioğlu and Ergene (2006) investigated EA on the hunger and shenmen points of the auricle and found that the points LI4, LI11, ST36, and ST44 can alter the levels of biochemistry in obese women. The results revealed increased serum insulin and C-peptide levels in the EA group compared with the placebo group. A higher C-peptide level is positively correlated with a higher body mass index (BMI) [124]. C.-H. Yeh and S.-C. J. Yeh (2008) investigated obesity-related parameters in nonobese and obese participants after AA treatment. The results revealed a significant reduction in waist circumference (WC) and hip circumference (HC) in nonobese subjects, but not in obese subjects [125]. Shen et al. (2009) described another mechanism, which differed from the involvement of NPY reduction. After 4 weeks of AA treatment, their body weight decreased in both the AA group and the control group. Sympathomimetic effects were noted in both groups and the effects are increasing the basal metabolism and reducing appetite temporarily [126]. Hsu et al. (2009) monitored the effects of AA on obesity-related parameters and hormone peptides. The findings indicated no change in body weight, BMI, and WC between both groups, but a significant increase in ghrelin and a decrease in leptin in the AA group [127]. Hsieh (2010) investigated the effectiveness of AA on body weight and serum lipid levels in obese adolescents. The BMI was significantly decreased in all groups but the total cholesterol, triglycerides, high density lipids, and low density lipids were all significantly increased [128]. Ching et al. (2012) recruited 86 obese patients with schizophrenia and randomly assigned them to an AA group and a control group. No differences were found between the AA group and the control group in body weight, WC, and body fat percentage after an 8-week intervention [129]. Abdi et al. (2012) hypothesized that the effects of AA on the reduction in body weight were related to the immune system or the inflammatory process. The results revealed a reduction in anthropometric factors and antiheat shock protein antibodies, but not in high-sensitivity C-reactive protein levels in the AA group, and indicated that the effects of AA are induced through immunomodulation [130]. Yeo et al. (2014) investigated the effect of different auricular acupoints in improving obesity. The results revealed significant differences in BMI, body weight, and body fat percentage between the treatment and control groups, but no differences between the five-point (i.e., shenmen, spleen, stomach, hunger, and endocrine) and one-point (hunger) groups [131]. Darbandi et al. (2014) examined the effects of different acupuncture methods on abdominal fat reduction. Body EA and AA were both found to have significantly reduced BMI, WC, HC, and trunk fat mass. Body EA was more effective in reducing WC compared with AA, whereas AA had a greater effect in reducing HC [132]. Kim et al. (2014) examined the effects of AA combined with* Sinapis alba* seeds in treating obesity. They found a significant decrease in body weight and BMI in the AA group, but the changes in body fat percentage and waist-to-hip ratio were nonsignificant [118]. Set et al. (2014) investigated the effects of AA in treating depression in obese women. The results revealed that the BMI and Beck Depression Inventory for Primary Care scores decreased following AA treatment [133]. He et al. (2012) designed a randomized controlled clinical trial to compare the effect between both auricular acupressure and exercise and exercise alone on obesity. They found that both auricular acupressure and exercise for 4 weeks may produce greater effect than exercise alone for body weight reduction in Chinese women with primary obesity [134].

## 5. Conclusion

Auricular therapy is a convenient approach for treating diseases in areas lacking medical resources. Evidence on auricular therapy supports its efficacy for pain relief, in treating epilepsy and anxiety, as well as obesity, and in improving sleep quality, but not for treating substance dependence. The mechanism of auricular therapy warrants further study.
